# Applications of next-generation sequencing analysis for the detection of hepatocellular carcinoma-associated hepatitis B virus mutations

**DOI:** 10.1186/s12929-018-0442-4

**Published:** 2018-06-02

**Authors:** I-Chin Wu, Wen-Chun Liu, Ting-Tsung Chang

**Affiliations:** 1 Department of Internal Medicine, National Cheng Kung University Hospital, College of Medicine, National Cheng Kung University, 138 Sheng-Li Road, Tainan, 70403 Taiwan, Republic of China; 2 Infectious Disease and Signaling Research Center, National Cheng Kung University, Tainan, Taiwan, Republic of China

**Keywords:** Deletion mutation, Next-generation sequencing, Hepatitis B virus, Hepatocarcinogenesis, Hepatocellular carcinoma, Single-nucleotide variant, U-shaped distribution

## Abstract

**Background:**

Next-generation sequencing (NGS) is a powerful and high-throughput method for the detection of viral mutations. This article provides a brief overview about optimization of NGS analysis for hepatocellular carcinoma (HCC)-associated hepatitis B virus (HBV) mutations, and hepatocarcinogenesis of relevant mutations.

**Main body:**

For the application of NGS analysis in the genome of HBV, four noteworthy steps were discovered in testing. First, a sample-specific reference sequence was the most effective mapping reference for NGS. Second, elongating the end of reference sequence improved mapping performance at the end of the genome. Third, resetting the origin of mapping reference sequence could probed deletion mutations and variants at a certain location with common mutations. Fourth, using a platform-specific cut-off value to distinguish authentic minority variants from technical artifacts was found to be highly effective. One hundred and sixty-seven HBV single nucleotide variants (SNVs) were found to be studied previously through a systematic literature review, and 12 SNVs were determined to be associated with HCC by meta-analysis. From comprehensive research using a HBV genome-wide NGS analysis, 60 NGS-defined HCC-associated SNVs with their pathogenic frequencies were identified, with 19 reported previously. All the 12 HCC-associated SNVs proved by meta-analysis were confirmed by NGS analysis, except for C1766T and T1768A which were mainly expressed in genotypes A and D, but including the subgroup analysis of A1762T. In the 41 novel NGS-defined HCC-associated SNVs, 31.7% (13/41) had cut-off values of SNV frequency lower than 20%. This showed that NGS could be used to detect HCC-associated SNVs with low SNV frequency. Most SNV II (the minor strains in the majority of non-HCC patients) had either low (< 20%) or high (> 80%) SNV frequencies in HCC patients, a characteristic U-shaped distribution pattern. The cut-off values of SNV frequency for HCC-associated SNVs represent their pathogenic frequencies. The pathogenic frequencies of HCC-associated SNV II also showed a U-shaped distribution. Hepatocarcinogenesis induced by HBV mutated proteins through cellular pathways was reviewed.

**Conclusion:**

NGS analysis is useful to discover novel HCC-associated HBV SNVs, especially those with low SNV frequency. The hepatocarcinogenetic mechanisms of novel HCC-associated HBV SNVs defined by NGS analysis deserve further investigation.

## Background

Hepatitis B virus (HBV) is a serious health problem because patients with chronic HBV infection are at risk for development of liver cirrhosis and hepatocellular carcinoma (HCC). It is estimated that 240 million people are chronic HBV carriers worldwide and 15 to 40% of them will develop liver cirrhosis, liver failure, or HCC during their lifetime [1–5].

HBV is classified into ten genotypes, labeled A through J, and over 40 related sub-genotypes. The ten genotypes are based on an intergroup divergence of at least 8% in the complete nucleotide sequence, while the sub-genotypes are based on a 4 to 7.5% divergence [6, 7]. The ten genotypes are also commonly found in certain geographic locations as followed. Genotype A is the predominant genotype in Northern Europe and the United States. Genotypes B and C are common in East and Southeast Asia, while Genotype D is prevalent in the Mediterranean, Middle East, and South Asia. Genotype E has been reported in West Africa, genotype F in Central and South America, genotype G in the United States, France, and Germany, genotype H in Central America, genotype I in Vietnam, and genotype J in the Ryukyu Islands of Japan [8, 9]. It is important to note that HBV genotype A and B are associated with earlier hepatitis B e antigen (HBeAg) seroconversion, less active liver disease, and a slower rate of progression to liver cirrhosis and HCC as compared to HBV genotype C and D [9–12].

Naturally occurring mutations in the precore and basal core promoter (BCP) regions are common. The most common precore mutations are G1896A and G1899A, of which G1896A creates a stop codon and prevents the synthesis of HBeAg [13]. The most common BCP mutations are A1762T and G1764A, which are associated with reduced synthesis of HBeAg by suppressing the transcription of precore mRNA [14, 15]. The precore and BCP mutants are usually found in HBeAg-negative patients but could also present as a mixture with wild-type virus in HBeAg-positive patients [16, 17]. The precore mutations are more common in patients with HBV genotype B and D than in patients with HBV genotype A and C, whereas the BCP mutations are more common in patients with HBV genotype A and C than in patients with HBV genotype B and D [9, 18–21]. The precore and BCP mutants are associated with liver cirrhosis, HCC, and advanced liver disease [19, 22–25].

The pre-S protein plays an important role in the interaction with the immune system, as it contains B-cell and T-cell epitopes [26–28]. The pre-S1 domain contains the hepatocyte binding site and is essential for virion assembly and transportation [29–31]. The pre-S2 domain can bind to polymerized human serum albumin, but the significance of this binding is unknown [32]. The pre-S deletion mutations are prevalent in patients with chronic HBV infection, ranging from 6% at age 20–29 years to 35% at age 50–59 years in HBeAg-positive patients, and 60% in HCC patients [33]. These deletion mutations are found more frequent in genotype B (25%) and genotype C (24.5%) than in the other genotypes [34]. Some studies showed that pre-S deletion mutations are an independent risk factor for HCC [35–37], while other studies showed that combination of mutations (pre-S deletion, precore, and BCP mutations) rather than a single mutation, are associated with liver cirrhosis and liver diseases progression [38, 39]. Pre-S deletion mutations could induce endoplasmic reticulum (ER) stress, genomic instability, and hepatocyte proliferation [40–43]. In the transgenic mouse model, the pre-S2 deletion mutations can induce dysplasia of hepatocytes and HCC development [33, 44].

Hepatitis B virus X protein (HBx), a nonstructural protein, is required for HBV covalently closed circular DNA (cccDNA) transcription and viral replication [45, 46]. In addition, HBx contributes to hepatocarcinogenesis through interactions with multiple cellular proteins that modulate cell proliferation, cell death, gene expression, and DNA repair [47–50]. Truncated HBx proteins have also been reported to promote hepatocarcinogenesis [51, 52].

Previous studies showed that the risk of HCC was associated with the existence of specific HBV variants, which were the major stains identified by traditional direct Sanger sequencing. Although direct Sanger sequencing is the most common method for analyzing viral mutations, it is unable to determine the profile of a heterogeneous viral population in a patient. Next-generation sequencing (NGS) however, can do this, as well as perform high-throughput analysis from thousands of amplified regions, characterize genetic diversity, and detect minor strains that direct sequencing or cloning neither can find [53–55]. This study provides an overview about the possible applications of next-generation sequencing analysis for the detection of hepatocellular carcinoma-associated hepatitis B virus mutations.

### Optimization of NGS analysis for HBV: Four recommended steps

#### Use the sample-specific reference sequence as the mapping reference

Assembling the NGS reads into whole-genome sequences could be performed by de novo assembly or mapping using reference sequences. De novo assembly is usually employed in studying unknown species and would be hindered by regions with high diversity. For studying HBV, mapping reference is often utilized [55–57]. Two main NGS platforms, Illumina Genome Analyzer and Roche Genome Sequencer, were widely used in viral quasispecies studies. Illumina generates larger data sets with shorter read length, as compared with Roche. Therefore, the NGS data generated by Illumina are usually assembled using reference sequences as templates while de novo assembly is applicable but not commonly used [58, 59].

One of the major challenges for NGS is to monitor quality control metrics over all stages of the data processing pipeline. Alignment with a reconcilable mapping reference is a required step for any re-sequencing analysis and is crucial for successful variant detection [60].

HBV quasispecies involves an error-prone reverse transcription step in its replication, so that its rate of nucleotide change during replication is high and closed to the rate observed for the RNA viruses. The evolution rate of HBV ranges from 1.8 × 10^− 2^ to 1.5 × 10^− 5^ nucleotide substitutions/site/year [61–63], while that of the human genome is 1.1–3 × 10^− 8^ nucleotide substitutions/site/generation [64]. Furthermore, HBV has differences in genomic lengths among 10 HBV genotypes (from 3182 to 3248 base pairs), which could result in genotype alignments containing several regions of gaps [65].

**Fig. 1:**
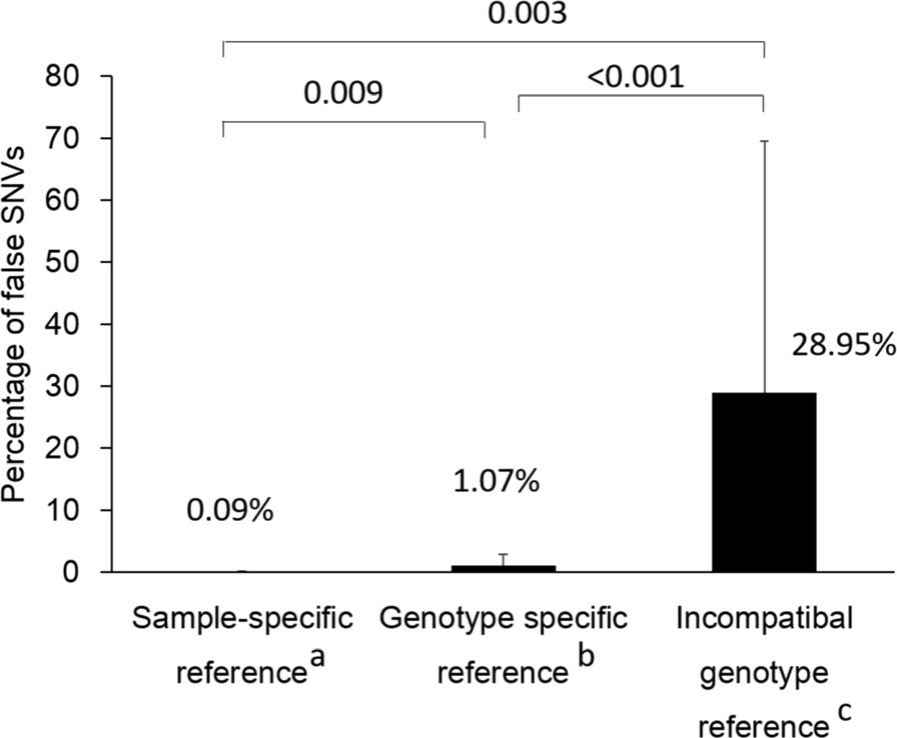
The percentage of false SNV calls for using a different reference sequence. Full-length HBV genome sequence, Clone_N6 (KJ790199; genotype C, Taiwan) was cloned from a CHB patient and sequenced using a direct Sanger sequencer. This nucleotide sequence would be used as a standard sequence. Clone_N6 was also fragmented to be sequenced by NGS analysis. The mapping results of NGS reads from the Clone_N6 using the following mapping references: sample-specific reference, genotype specific reference (JN315779; genotype C, Asia) and incompatible genotype reference (FJ787477; genotype B, Asia). When compared with the standard sequence of Clone_N6, derived from direct sequencing, the percentage of false SNVs calls increased significantly from 0.09% using sample-specific reference as mapping reference to 28.95% using incompatible genotype reference as the mapping reference. ^a^Sample-specific reference is the consensus sequence obtained from the NGS reads of each sample through alignment with its same genotype mapping reference. ^b^Reference is using the same genotype as the sample (genotype C). ^c^Reference is using the incompatible genotype as the sample (genotype B)

Previous HBV-related NGS analyses used the consensus genotype sequences from public viral databases [55, 56] or the major viral sequence identified by polymerase chain reaction (PCR)-director sequencing [57] as mapping references to detect HBV variants. A sample-specific reference sequence is the consensus sequence obtained from the NGS reads of each sample through alignment with its same genotype mapping reference. In our demonstrations, we found that using this type of reference sequence as the mapping reference has the best mapping quality and the highest single nucleotide variant (SNV) calling accuracy, as compared with using the compatible genotype sequence [66]. The percentage of false SNV calls increased significantly from 0.09% using a sample-specific reference sequence to 28.95% using an incompatible genotype reference (Fig. 1). These false SNVs would be especially prone to call in regions with high divergence. In addition, the sample-specific reference sequence is effective in the analysis of HBV quasispecies, which is more complex to analyze due to its hetereogeneity and structure.

#### Elongate the end of reference sequence and reset the origin of mapping reference sequence

HBV genome is a circular structure with position 1 conventionally taken to be the first “T” nucleotide in the EcoR1 restriction site (“GAATTC”) [67]. Some variants and deletion mutations, such as pre-S deletion mutations, cross this site. Most genome mappers for NGS analysis, like BWA [12], were designed for linear genome, but they were not well suited for circular genomes like HBV genomes and will have worse mapping performance when reads spanned the end of genome. To resolve the problem, we manually concatenated the end of reference sequence for 600 bases and reset the origin of mapping reference sequence from nt1600. This approach was beneficial to improve mapping performance at the end of genomic sequence and detect deletion mutations spanning position 1 of HBV genome [68].

#### Use a platform-specific cut-off value to distinguish authentic minority variants from technical artifacts

High-throughput sequencing techniques can generate low-interest variants in the form of false-positives, especially from misalignment of sequencing reads and inaccuracies of the reference sequence compared to a specific local population [69]. In order to distinguish authentic minority variants from technical artifacts, we estimated the technical error rate and identified a threshold above which mutations detected by NGS using Illumina HiSeq™ 2500 were unlikely to be technical artifacts. The technical error rate was estimated by PCR amplification and NGS of a plasmid expressed with HBV full-length genome. The mean error rate among three runs was estimated by comparing each NGS sequence read to the plasmid control sequences. The empirical distribution of mismatch and deletion errors in the clone yielded an average of 0.32 and 1.8%, respectively. Accordingly, we used this empirically observed distribution of mismatch errors to distinguish sequence errors from authentic minor variants by excluding possible technical errors, which were mutations present in < 3.2% of sequence reads, a value 1 log above the mean overall error rate in the Illumina HiSeq™ 2500 platform. For deletion mutations, an exclusionary cutoff of < 1.8% was used [66, 68]. Some other studies had proposed the similar approach to distinguish authentic minority variants from technical artifacts with different cut-off value in its current platform [55–57]. This is an important step not to be ignored after variant calling.

#### Apply these two analytic methods to better identify the deletion mutations in the HBV genome

Higher heterogeneity increases the uncertainty of reads-mapped genomic coordinates and leads to greater challenges in discovering deletion mutations. Several methods for deletion mutation discovery have been proposed, such as BreakDancer [70], Pindel [71], Breakpointer [72], but all these tools were mainly designed for human NGS data and not entirely applicable for viruses with a high mutation rate. DeF-GPU is a graphics processing unit-based data mining method that incorporates the pattern growth approach to identify HBV genomic deletions. Validation of DeF-GPU on synthetic and real datasets showed that DeF-GPU outperforms the representative and commonly-used method Pindel, a pattern growth approach originally designed to detect either large deletions or medium-sized insertions, and is able to exactly identify the deletions in few seconds [73]. VirDelect uses the split read alignment method to obtain the exact breakpoints of deletions. The experiments on simulation data and real data indicated that VirDelect can identify more exact breakpoints of deletions than Pindel and is suitable for researchers with higher requirements in accuracy than speed [74].

### HCC-associated HBV SNVs determined by next-generation sequencing analysis

Through HBV genome-wide NGS analysis, our previous study identified 60 NGS-defined HCC-associated SNVs and their pathogenic frequencies, including 41 novel SNVs. Each SNV was specific for either genotype B (*n* = 24) or genotype C (*n* = 34), except for nt53C, which was identified in both genotypes. SNV I was defined as the dominant strain of HBV in the majority of non-HCC patients. SNV II was defined as the variant other than SNV I at the same nucleotide position, i.e. the minor strain of HBV in the majority of non-HCC patients [68].

#### HCC-associated HBV SNVs for genotype B

**Fig. 2:**
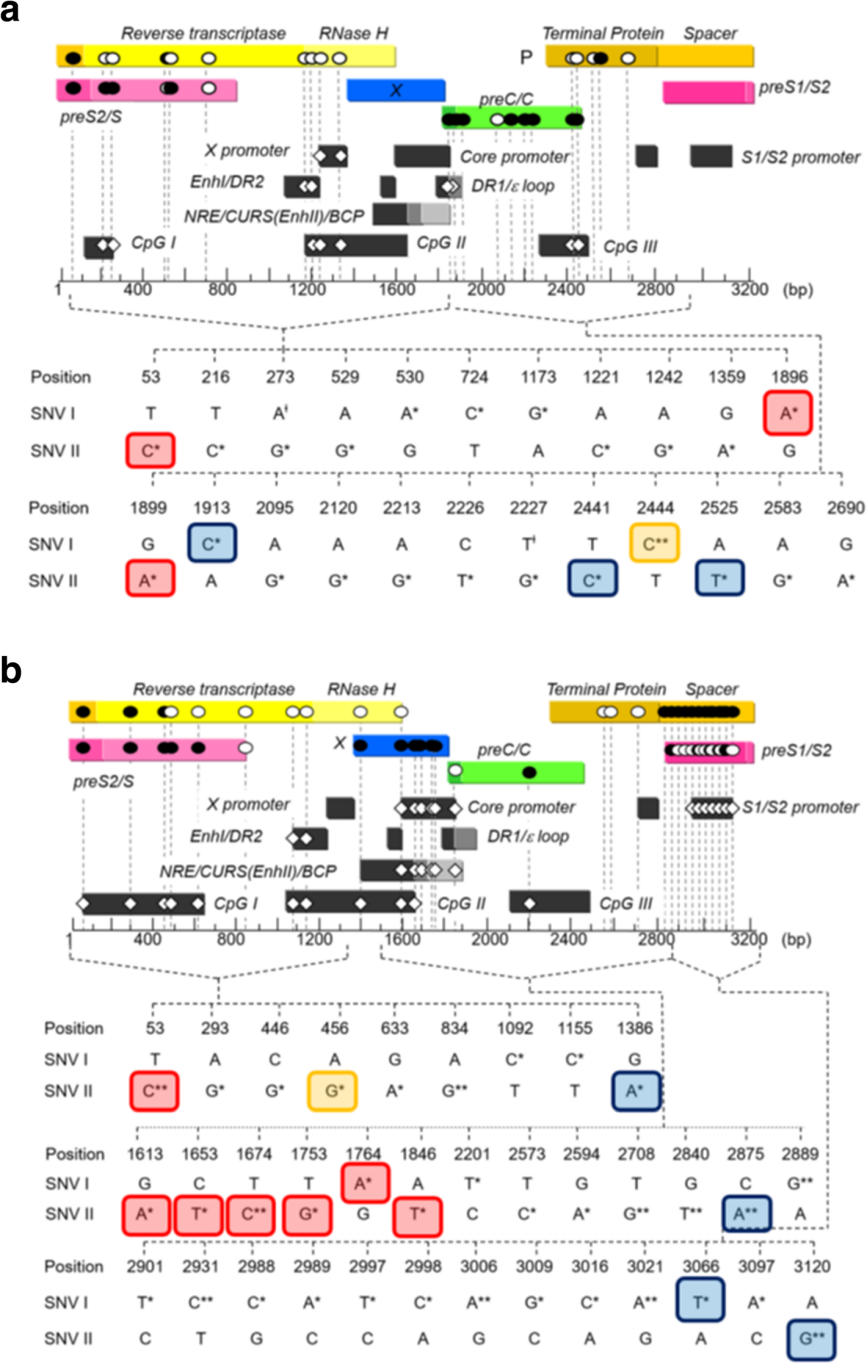
Distinct NGS-derived SNVs located in HBV regulatory element and ORFs associated with HCC among patients with genotype B and genotype C. **a**, Twenty-five distinct NGS-defined HCC-associated SNVs were located in HBV regulatory elements and ORFs for genotype B HBV. **b**, Thirty five distinct NGS-defined HCC-associated SNVs were located in HBV regulatory elements and ORFs for genotype C HBV. * and ** indicate risk of SNVs for HCC with an odds ratio of HCC > 1 and with *P* value of < 0.05 and < 0.01, respectively. Ɨ means protective SNVs for HCC with an odds ratio of HCC < 1 and with a *P* value < 0.05. ● missense mutation; ○ silent mutation; • SNVs located in regulatory element. Level A means HCC-associated HBV variants supported by meta-analysis with at least 4 studies. Level B means HCC-associated HBV variants supported by at least one study if total number of relevant studies is less than 4. Level C means HBV variants unassociated with HCC supported by all studies if total number of relevant studies is less than 4. Red box indicated Level A; Blue box indicated Level B; Yellow box indicated Level C. The figure has been adopted from [70]

For genotype B, 25 HCC-associated SNVs located at 23 nucleotide sites were identified, including the precore mutations (G1896A and G1899A). For nucleotide sites 273 and 2227, 273A and 2227 T were SNV I and protective factors for HCC, whereas 273G and 2227G were SNV II and risk factors for HCC. All the other 21 SNVs were risk factors for HCC, 6 of them were SNV I and 15 of them were SNV II (Table 1). Seventeen of 25 SNVs were missense mutations at the polymerase, preS2, surface, precore, and core regions. Seven of the 17 missense mutations and 4 of the 8 silent mutations were at the regulatory elements, including CpG islands I/II/III, X promoter, enhancer (Enh) I, ε loop, and BCP (Fig. 2).

**Table 1:** HCC-associated SNVs with their pathogenic frequencies through NGS analysis, categorized by level of supporting evidence

Genotype B				Genotype C			
Site	NT	Odds ratio (95% CI)	Pathogenic frequency (%)	Site	NT	Odds ratio (95% CI)	Pathogenic frequency (%)
Level A							
53	C	4.5 (1.8, 11.5)	32	53	C	5.1 (1.6, 16.7)	52.4
1896	A	3.5 (1.1,10.7)	96.5	1613	A	10.1 (1.2, 83.6)	86.7
1899	A	5.8 (1.1, 31.0)	96.1	1653	T	4.2 (1.3, 14.2)	60.8
				1674	C	11.5 (1.4, 94.8)	55.8
				1753	G	3.1 (1.1, 9.4)	100
				1764	A	4.6 (1.7, 12.4)	100
				1846	T	3.1 (1.2, 8.2)	21.1
Level B							
1913	C	5.5 (1.1, 28.2)	94.3	1386	A	5.2 (1.1, 25.9)	96.7
2441	C	11.5 (1.4, 96.5)	100	2875	A	5.6 (1.7, 18.3)	94.8
2525	T	7.5 (1.5, 36.7)	95.5	3066	T	5.6 (1.2, 26.6)	86.8
				3120	G	4.8 (1.6, 14.4)	95.9
Level C							
2444	C	4.8 (1.6, 14.3)	92.6	456	G	6.0 (1.2, 29.3)	10.2
Novel							
216	C	5.6 (1.2, 28.3)	100	293	G	2.9 (1.3, 6.4)	1.8
273	G	5.0 (2.0, 12.8)	12.2	446	G	5.6 (2.0, 15.2)	6.4
273	A	0.3 (0.1, 0.8)	55	633	A	5.6 (1.1, 27.5)	3.6
529	G	4.1 (1.4, 12.0)	100	834	G	7.6 (2.1, 28.2)	76.9
530	A	4.2 (1.3, 14.0)	100	1092	C	5.6 (1.2, 26.6)	50.1
724	C	4.0 (1.3, 12.0)	91.7	1155	C	5.6 (1.2, 26.6)	87.1
1173	G	9.2 (1.1, 76.5)	94.1	2201	T	3.7 (1.1, 11.9)	93.3
1221	C	4.1 (1.1, 14.1)	4.3	2573	C	4.2 (1.3, 14.2)	100
1242	G	5.1 (1.8, 14.6)	5.5	2594	A	4.0 (1.3, 12.4)	100
1359	A	3.6 (1.0, 12.5)	2.4	2708	G	5.8 (1.5, 22.1)	100
2095	G	3.2 (1.3, 8.0)	10.1	2840	T	8.3 (1.8, 38.3)	4.4
2120	G	3.6 (1.0, 12.5)	67.2	2889	G	5.5 (1.8, 17.6)	85.3
2213	G	9.8 (1.2, 83.1)	1.7	2901	T	4.1 (1.1, 15.4)	85.1
2226	T	9.8 (1.2, 83.1)	1.7	2931	C	9.1 (2.0, 41.6)	87.1
2227	G	9.8 (1.2, 83.1)	1.8	2988	C	9.0 (1.1, 73.6)	92.1
2227	T	0.2 (0.1, 0.8)	95.5	2989	A	9.0 (1.1, 73.6)	91.7
2583	G	6.5 (1.2, 32.3)	100	2997	T	9.0 (1.1, 73.6)	90.8
2690	A	4.9 (1.2, 19.3)	12	2998	C	9.0 (1.1, 73.6)	92.2
				3006	A	14.1 (1.8, 111.8)	94.2
				3009	G	5.0 (1.0, 24.0)	92.9
				3016	C	9.0 (1.1, 73.6)	92.9
				3021	A	11.4 (1.4, 91.9)	92.6
				3097	A	4.5 (1.2, 16.8)	93.5

Note: The pathogenic frequencies were the cut-off values of SNV frequency for HCC-associated SNVs. The table has been adopted from [70]

#### HCC-associated HBV SNVs for genotype C

For genotype C, all the 35 HCC-associated SNVs located at distinct nucleotide site were found, including BCP mutations (G1764A and C1653T). All the 35 SNVs were risk factors for HCC, 17 of them were SNV I and 18 of them were SNV II (Table 1). Twenty-eight of 35 SNVs were missense mutations located at 4 open reading frames (ORFs), particularly at the preS1 region and the spacer domain of polymerase. Twenty one of the 28 missense mutations and 3 of the 6 silent mutations were at the regulatory elements, including CpG islands I/II/III, negative regulatory element (NRE)/core upstream regulatory sequence (CURS)/BCP, Enh I/II, core promoter, and S2 promoter (Fig. 2).

#### The U-shaped distribution pattern of SNV frequency in SNV II and the novel HCC-associated SNVs with low SNV frequency detected by NGS analysis

**Fig. 3:**
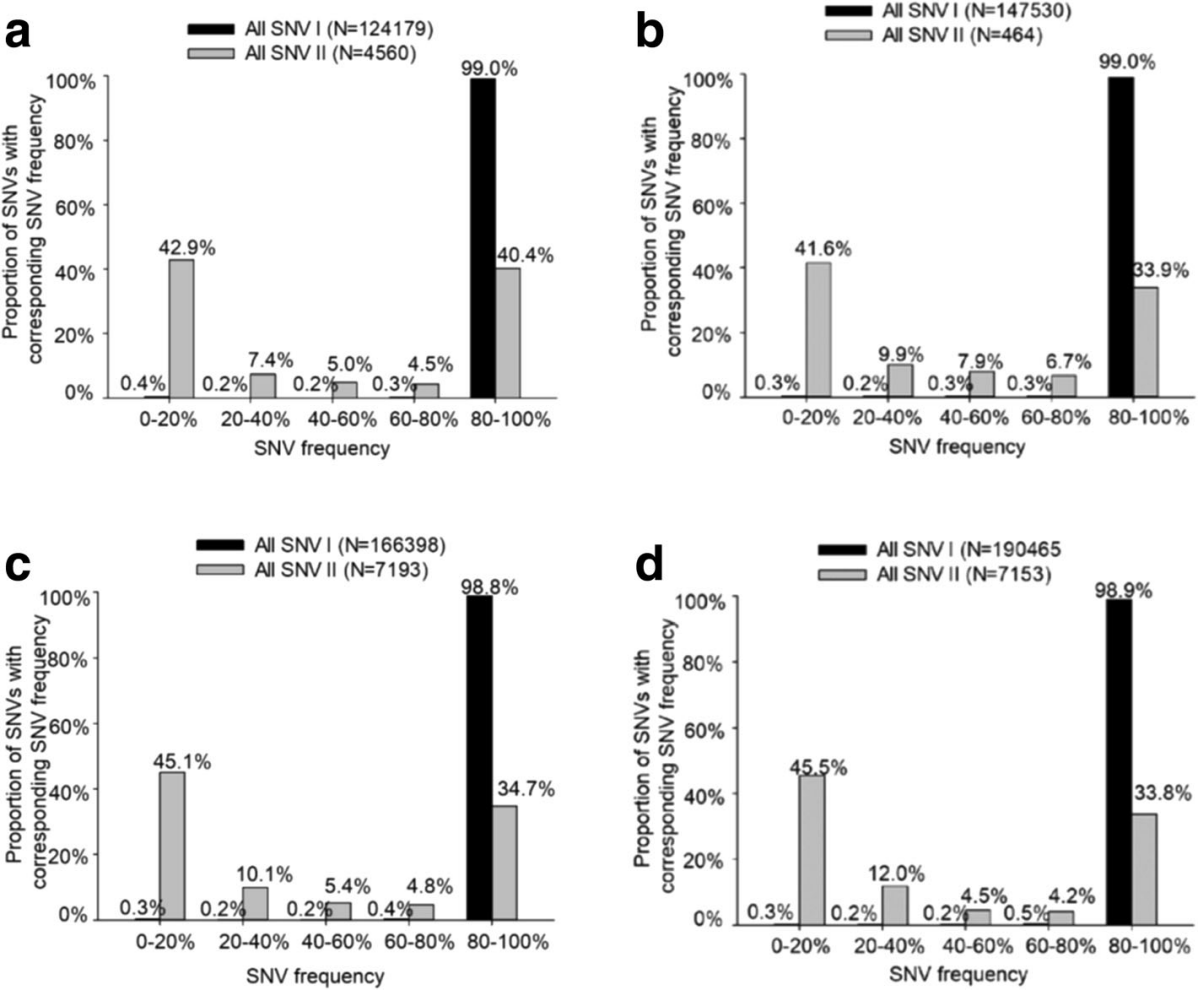
The distribution of SNV frequencies in SNV I and SNV II. Almost all SNV I had SNV frequencies higher than 80%. The great majority of SNV II had either low (< 20%) or high (> 80%) SNV frequencies, i.e. a characteristic U-shaped distribution pattern of SNV frequencies with low (< 20%) or high (> 80%) values. **a**, All SNVs in genotype B HCC group. **b**, All SNVs in genotype B non-HCC group. **c**, All SNVs in genotype C HCC group. **d**, All SNVs in genotype C non-HCC group. SNV I was defined as the dominant strain of HBV in non-HCC group. SNV II was defined as the variant other than SNV I at the same nucleotide position, i.e. the minor strain of HBV in non-HCC group

**Fig. 4:**
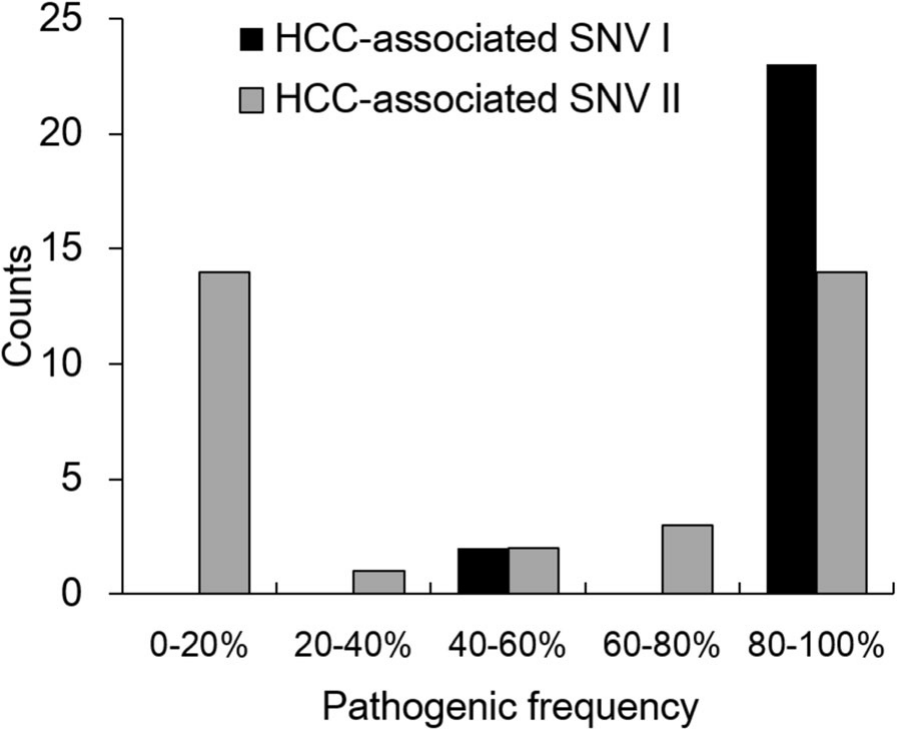
The distribution of pathogenic frequencies in HCC-associated SNV I and SNV II. Almost all HCC-associated SNV I had pathogenic frequencies higher than 80% and the great majority of HCC-associated SNV II had either low (< 20%) or high (> 80%) pathogenic frequencies, i.e. a U-shaped distribution pattern. SNV I was defined as the dominant strain of HBV in non-HCC group. SNV II was defined as the variant other than SNV I at the same nucleotide position, i.e. the minor strain of HBV in non-HCC group

**Fig. 5:**
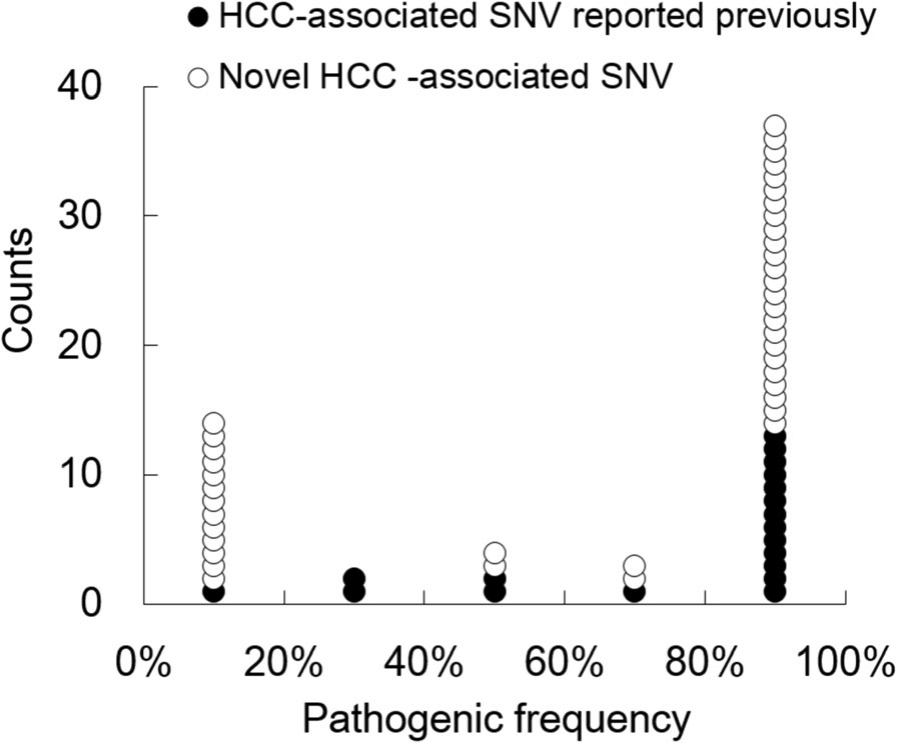
The distribution of pathogenic frequencies in previously reported and novel HCC-associated SNVs. Among the 60 NGS-defined HCC-associated SNVs, 19 had been reported previously and 41 were novel ones. In 19 HCC-associated SNVs reported previously, 94.7% (18/19) had cut-off values of SNV frequency > 20%, expect nt456G, which had a cut-off value of 10.2%. In the other 41 novel HCC-associated SNVs, 68.3% (28/41) had cut-off values of SNV frequency > 20 and 31.7% (13/41) had cut-off values < 20%

Almost all SNV I had SNV frequencies higher than 80%. The great majority of SNV II had either low (< 20%) or high (> 80%) SNV frequencies, i.e. a characteristic U-shaped distribution pattern of SNV frequencies with low (< 20%) or high (> 80%) values (Fig. 3). The cut-off values of SNV frequency for HCC-associated SNVs represent their pathogenic frequencies. Almost all HCC-associated SNV I had pathogenic frequencies higher than 80% and the great majority of HCC-associated SNV II had either low (< 20%) or high (> 80%) pathogenic frequencies, a U-shaped distribution pattern (Fig. 4). Among the 60 NGS-defined HCC-associated SNVs, 19 had been reported previously and 41 were novel ones. In 19 HCC-associated SNVs reported previously, 94.7% (18/19) had cut-off values of SNV frequency greater than 20%, except nt456G, which had a cut-off value of 10.2%. In the other 41 novel HCC-associated SNVs, 68.3% (28/41) had cut-off values of SNV frequency greater than 20%, while 31.7% (13/41) had cut-off values of less than 20% (Fig. 5). This showed that NGS could be used to detect HCC-associated SNVs with low SNV frequency.

#### Validation of the NGS-defined HCC-associated SNVs

For validating the 60 NGS-defined HCC-associated SNVs, a systematic literature review and meta-analysis was conducted. One hundred and sixty-seven HBV variants had been studied previously and were categorized into 4 levels of supporting evidence associated with HCC. Level A included 12 HCC-associated HBV variants supported by meta-analysis with at least 4 studies. Level B included 60 HCC-associated HBV variants supported by at least one study if total number of relevant studies were less than 4. Level C included 85 HBV variants unassociated with HCC supported by all studies if total number of relevant studies were less than 4. Level D included 10 HBV variants unassociated with HCC supported by meta-analysis with at least 4 studies. The proportions of NGS-defined HCC-associated SNVs among HBV variants with different levels of supporting evidence declined significantly with decreasing levels of evidence from Level A to Level D. All the HCC-associated HBV variants with Level A evidence, except for C1766T and T1768A which were mainly expressed in genotypes A and D, and the subgroup analysis of A1762T, were identified by NGS analysis. Besides, 5 novel NGS-defined HCC-associated SNVs in the small surface region identified by our previous study did influence hepatocarcinogenesis pathways, including endoplasmic reticulum-stress and DNA repair systems, as shown by microarray, real-time polymerase chain reaction and western blot analysis [68].

#### The advantage of NGS for the detection of HCC-associated HBV mutations

Our previous NGS analysis showed that the association of HCC was related to specific SNVs and deletion mutations with a certain frequency instead of presence or absence of specific variant. Risk HCC-associated SNVs had significantly higher SNV frequency in HCC group than in non-HCC group, whether they were dominant strains or minor strains in HCC group. Protective HCC-associated SNVs had significantly lower SNV frequency in HCC group than in non-HCC group. For deletion mutations, the deletion of preS region was significantly associated with HCC, in terms of deletion index, which is composed of the deletion length and the deletion frequency by NGS analysis, but there was no significant difference in the proportions of patients with deletion mutations between HCC patients and non-HCC patients. In addition, the lower limit of detection using direct Sanger sequencing technology is ~ 20% minor allele frequency. In our previous study, 31.7% (13/41) novel HCC-associated SNVs and 83.6% (138/165) deletion mutations had cut-off values of SNV frequency lower than 20%, which could only be detected by NGS analysis [68]. Therefore, NGS is a powerful tool to characterize minor strains among viral quasispecies which could not be detected even by direct sequencing or cloning.

### HBV SNVs and deletion mutations related to HCC development

The mechanisms of hepatocarcinogenesis induced by HBV quasispecies are still not completely known. Many studies had indicated that unique HBV oncoproteins (HBx isoforms and preS mutants) and mutated precore/core proteins could induce hepatocarcinogenesis through induction of endoplasmic reticulum (ER) and oxidative stress [40], activation of ER-independent pathway [75], regulation of microRNA expression [76], lipid metabolism disturbance [77], or epigenetic modification through modified genomic methylation status [78]. Mutations of HBV regulatory elements probably induced hepatocarcinogenesis through oncoprotein expression modulation [79, 80], HBV DNA integration leading to chromosomal instability [81], or HBV DNA methylation [82].

#### Surface gene and protein

The HBV surface (S) proteins are produced from ORF S gene with three different translation sites, pre-S1, pre-S2, and S, to large, middle, and small surface proteins. The variability of the pre-S1 and pre-S2 regions were higher in the HCC group than in the non-HCC group [68, 83], and the mutations at the promoter sites of pre-S1 and pre-S2 were significantly associated with an increased risk of HCC [37, 84]. The pre-S mutated large surface protein are retained in the ER to induce ER stress signals and upregulate COX-2 and cyclin A to induce cell cycle progression [33]. According to our previous NGS analysis for pre-S deletion in genotype C HBV, the HCC group had more patients with deletion mutations involving nt2977–3013 (amino acid 43–56), deletion patterns II or III [38], deletion mutations at S2 promoter, and heat shock protein binding site in the preS region than the non-HCC group [68]. Pre-S deletion mutants can cause accumulation of HBsAg in the ER and lead to ER stress and oxidative stress, which is known to cause DNA damage and alterations of several signaling pathways that are related to cell proliferation, invasion, cell survival, and apoptosis [85].

In addition, a few point mutations of HBV surface proteins were reported to be associated with HCC, such as Q10L in pre-S1 region [86], F22 L in pre-S2 region [86], and I126S, G130 N, M133 L/T, and G145R in S region [86–89]. However, the hepatocarcinogenesis mechanisms of these variants remain unclear. From our previous NGS results, 4 missense mutations and 2 silent mutations located in ORF S gene of genotype B HBV and concentrated on pre-S2 and small S regions; 10 missense mutations and 11 silent mutations distributed in ORF S gene of genotype C HBV. We showed again that amino acid F22 L (nt T53C), Q10L (nt C2875A) and A216T (nt G530A) were HCC-associated variants. On the other hand, we also identified the other NGS-defined HCC-associated SNVs in small S region (Genotype B, nt T216C and nt A273G; Genotype C, nt A293G, nt C446G, and nt A456G) could affect hepatocarcinogenesis pathway through inducing ER stress and regulating DNA repair system [68].

#### X gene and protein

HBx, a protein encoded by HBV ORF X gene, was involved in many intracellular signal pathways which were closely associated with cell proliferation and cell apoptosis [85]. Different HBx isoforms and C-terminal truncated HBx play important roles in HCC development [85, 90]. HBx C-terminal region could interact with intracellular molecules, through phosphorylation/methylation or binding to certain molecules, which directly or indirectly contribute towards tumorigenesis [91–94]. The C-terminal truncation of HBx plays a role in enhancing cell invasiveness and metastasis in HCC, regulating miRNA transcription and promoting hepatocellular proliferation [95–97]. From our previous NGS analysis, HBx deletions occurred only in a minority of patients with HCC (genotype B: 3% (1/40), genotype C: 4% (2/53)) and non-HCC (genotype B: 4% (2/47), genotype C: 3% (2/61)), and were indeed localized in the C-terminal of HBx. However, the proportion of patients with C-terminal truncation of HBx did not differ between HCC and non-HCC patients.

HBx-Ser31, an HBx mutation, had been investigated to exercise as an anti-apoptotic protein, resulting in enhancing tumor growth and suppressing tumorigenesis [90]. Another study showed that HBV BCP mutations (A1762T/G1764A), harbored in HBx gene lead to L130 M and V131I substitutions, could enhance S-phase kinase-associated protein 2 transcription, conversely down-regulate cell cycle inhibitors, and provide a potential mechanism for HCC development [91]. The Combo (T1753A/A1762T/G1764A/T1768A) mutations in BCP result in four amino acid substitutions in HBx protein including I127R/S/T, L130 M, V131I, and F132Y, which cause constitutive activation of the *Wnt* signaling pathway and play a pivotal role in HBV-associated hepatocarcinogenesis [98]. According to our previous NGS results, genotype C HBV bear HCC-associated SNVs in X gene (G1386A, G1613A, C1653T, T1674C, T1753G, and G1764A) and most of them clustered on C terminal of HBx, while genotype B HBV did not. These mutations changed HBx protein sequences to 5 M, 80I, 94Y, 101P, 127S, and 131I, which might affect the regulatory domain to change the self-regulatory mechanism of X gene expression, and impact the transactivation domain to regulate HBV replication and cellular pathway [99–102].

#### Precore/core gene and protein

The BCP and its adjacent precore region are crucial for replication of HBV. HBV mutations at BCP and precore region have been considered classical risk factors for HBV-related HCC, such as T1753 V, G1896A, G1899A, G1613A, and C1653T, which occur in core promoter and ORF C gene [103–106]. The BCP A1762T/G1764A double mutations have been indicated to increase the risk of HCC development exclusively in genotype C, but not in genotype B [25]. For our previous NGS results, G1896A and G1899A were HCC-associated variants barely in genotype B, while G1613A, C1653T, T1674C, T1753 V, G1764A, and A1846T were genotype C specific HCC-associated variants. A1762T was identified as an HCC-associated SNV by our NGS-based subgroup analysis of HBeAg-positive patients with genotype C HBV infection. Based on our meta-analysis and NGS results, we again confirmed the mutations T1727A, A1752G, C1773A and C1799G at BCP region and that T1858C and G1862 T at ORF C gene were not the risk variants for HCC development [68]. Mutations in BCP and core gene were usually considered to possess the trans-activating effect to the core promoter, resulting from alteration of binding affinity with trans-activator [107]. These hotspot mutations then would influence the complicated changes in genomic activity for HBeAg expression and HBV DNA replication, which may possibly lead to a more active hepatitis and the risk to HCC [107, 108].

#### Polymerase gene and protein

The association between HBV polymerase (P) gene mutations and HCC has been rarely reported. HBV P gene contain 4 domains as follows: a terminal protein (TP) region involved in priming the viral template, a spacer (SP) region, a catalytic domain with reverse transcriptase (RT) activity, and a C-terminus that has ribonuclease H (RNase H) activity. Polymerase dysfunction, in the form of an inability to package pre-genomic RNA into core particles, appeared to result from a single missense mutation in the 5′ region of the gene in a single patient with HCC [109]. Focusing on RT domain which overlaps with S gene, Wu et al. had characterized spontaneous mutations in the HBV RT region and indicated that A799G, A987G, and T1055A were independent risk factors for HCC using Sanger sequencing [110]. Li et al. indicated that rtF221Y (T791A), identified by the Sanger method, was an independent risk factor for the postoperative recurrence of HCC and poor overall survival rates [111]. Regarding the HCC-associated SNVs by our previous NGS analysis, only rtN134D (nt A529G) and tpK93E (nt A2583G) of genotype B and rtH55R (nt A293G) and rtS106C (nt C446G) of genotype C were nonsynonymous substitution in TP and RT domains affecting viral replication fitness. The other SNVs, located in spacer region and overlapped with pre-S region, did not affect the polymerase activity [68]. The related mechanisms of these HCC-associated SNVs involved in polymerase activity and hepatocarcinogenesis need to be further explored.

## Conclusion

NGS analysis is a powerful and high-throughput method for the detection of HCC-associated HBV mutations. This method is useful to discover novel HCC-associated HBV SNVs, especially those with low SNV frequency. Although our previous study confirmed the association between hepatocarcinogenesis and some novel HCC-associated HBV SNVs with low SNV frequency in small S region, the pathologic and clinical significance of these low frequency SNVs should be investigated further. In addition, the evolution and impact of these quasispecies, including these SNVs, are intriguing to be investigated.

## Abbreviations

BCP: Basal core promoter
cccDNA: Covalently closed circular DNA
CURS: Core upstream regulatory sequence
Enh: Enhancer
ER: Endoplasmic reticulum
HBeAg: Hepatitis B e antigen
HBV: Hepatitis B virus
HBx: Hepatitis B virus X protein
HCC: Hepatocellular carcinoma
NGS: Next-generation sequencing
NRE: Negative regulatory element
ORF: Open reading frame
P: Polymerase
PCR: Polymerase chain reaction
RNaseH: Ribonuclease H
RT: Reverse transcriptase
S: Surface
SNV: Single nucleotide variant
SP: Spacer
TP: Terminal protein

## References

[1] Lavanchy D (2004). Hepatitis B virus epidemiology, disease burden, treatment, and current and emerging prevention and control measures. J Viral Hepat.

[2] El-Serag HB (2012). Epidemiology of viral hepatitis and hepatocellular carcinoma. Gastroenterology.

[3] Sarin SK, Kumar M, Lau GK, Abbas Z, Chan HL, Chen CJ (2016). Asian-Pacific clinical practice guidelines on the management of hepatitis B: a 2015 update. Hepatol Int.

[4] Terrault NA, Bzowej NH, Chang KM, Hwang JP, Jonas MM, Murad MH (2016). AASLD guidelines for treatment of chronic hepatitis B. Hepatology.

[5] Lampertico P, Agarwal K, Berg T, Buti M, Janssen HLA, Papatheodoridis G (2017). EASL 2017 clinical practice guidelines on the management of hepatitis B virus infection. J Hepatol.

[6] Kramvis A, Arakawa K, Yu MC, Nogueira R, Stram DO, Kew MC (2008). Relationship of serological subtype, basic core promoter and precore mutations to genotypes/subgenotypes of hepatitis B virus. J Med Virol.

[7] Sunbul M (2014). Hepatitis B virus genotypes: global distribution and clinical importance. World J Gastroenterol.

[8] Chu CJ, Keeffe EB, Han SH, Perrillo RP, Min AD, Soldevila-Pico C (2003). Hepatitis B virus genotypes in the United States: results of a nationwide study. Gastroenterology.

[9] Lin CL, Kao JH (2011). The clinical implications of hepatitis B virus genotype: recent advances. J Gastroenterol Hepatol.

[10] Chan HL, Hui AY, Wong ML, Tse AM, Hung LC, Wong VW (2004). Genotype C hepatitis B virus infection is associated with an increased risk of hepatocellular carcinoma. Gut.

[11] Chu CJ, Hussain M, Lok AS (2002). Hepatitis B virus genotype B is associated with earlier HBeAg seroconversion compared with hepatitis B virus genotype C. Gastroenterology.

[12] Sumi H, Yokosuka O, Seki N, Arai M, Imazeki F, Kurihara T (2003). Influence of hepatitis B virus genotypes on the progression of chronic type B liver disease. Hepatology.

[13] Akahane Y, Yamanaka T, Suzuki H, Sugai Y, Tsuda F, Yotsumoto S (1990). Chronic active hepatitis with hepatitis B virus DNA and antibody against e antigen in the serum. Disturbed synthesis and secretion of e antigen from hepatocytes due to a point mutation in the precore region. Gastroenterology.

[14] Gunther S, Piwon N, Iwanska A, Schilling R, Meisel H, Will H (1996). Type, prevalence, and significance of core promoter/enhancer II mutations in hepatitis B viruses from immunosuppressed patients with severe liver disease. J Virol.

[15] Gunther S, Piwon N, Will H (1998). Wild-type levels of pregenomic RNA and replication but reduced pre-C RNA and e-antigen synthesis of hepatitis B virus with C(1653) −> T, a(1762) −> T and G(1764) −> a mutations in the core promoter. J Gen Virol..

[16] Chu CJ, Keeffe EB, Han SH, Perrillo RP, Min AD, Soldevila-Pico C (2003). Prevalence of HBV precore/core promoter variants in the United States. Hepatology.

[17] Hadziyannis SJ, Vassilopoulos D (2001). Hepatitis B e antigen-negative chronic hepatitis B. Hepatology.

[18] Chan HL, Hussain M, Lok AS (1999). Different hepatitis B virus genotypes are associated with different mutations in the core promoter and precore regions during hepatitis B e antigen seroconversion. Hepatology.

[19] Chen CH, Lee CM, Hung CH, Hu TH, Wang JH, Wang JC (2007). Clinical significance and evolution of core promoter and precore mutations in HBeAg-positive patients with HBV genotype B and C: a longitudinal study. Liver Int.

[20] Nguyen MH, Keeffe EB (2008). Are hepatitis B e antigen (HBeAg)-positive chronic hepatitis B and HBeAg-negative chronic hepatitis B distinct diseases?. Clin Infect Dis.

[21] Yuen MF, Fung SK, Tanaka Y, Kato T, Mizokami M, Yuen JC (2004). Longitudinal study of hepatitis activity and viral replication before and after HBeAg seroconversion in chronic hepatitis B patients infected with genotypes B and C. J Clin Microbiol.

[22] Chen CH, Lee CM, Lu SN, Changchien CS, Eng HL, Huang CM (2005). Clinical significance of hepatitis B virus (HBV) genotypes and precore and core promoter mutations affecting HBV e antigen expression in Taiwan. J Clin Microbiol.

[23] Kao JH, Chen PJ, Lai MY, Chen DS (2003). Basal core promoter mutations of hepatitis B virus increase the risk of hepatocellular carcinoma in hepatitis B carriers. Gastroenterology.

[24] Liu CJ, Chen BF, Chen PJ, Lai MY, Huang WL, Kao JH (2006). Role of hepatitis B virus precore/core promoter mutations and serum viral load on noncirrhotic hepatocellular carcinoma: a case-control study. J Infect Dis.

[25] Yang HI, Yeh SH, Chen PJ, Iloeje UH, Jen CL, Su J (2008). Associations between hepatitis B virus genotype and mutants and the risk of hepatocellular carcinoma. J Natl Cancer Inst.

[26] Ferrari C, Cavalli A, Penna A, Valli A, Bertoletti A, Pedretti G (1992). Fine specificity of the human T-cell response to the hepatitis B virus preS1 antigen. Gastroenterology.

[27] Maeng CY, Ryu CJ, Gripon P, Guguen-Guillouzo C, Hong HJ (2000). Fine mapping of virus-neutralizing epitopes on hepatitis B virus PreS1. Virology.

[28] Park JH, Cho EW, Lee YJ, Shin SY, Kim KL (2000). Determination of the protective effects of neutralizing anti-hepatitis B virus (HBV) immunoglobulins by epitope mapping with recombinant HBV surface-antigen proteins. Microbiol Immunol.

[29] Bruss V (1997). A short linear sequence in the pre-S domain of the large hepatitis B virus envelope protein required for virion formation. J Virol.

[30] Poisson F, Severac A, Hourioux C, Goudeau A, Roingeard P (1997). Both pre-S1 and S domains of hepatitis B virus envelope proteins interact with the core particle. Virology.

[31] Pontisso P, Ruvoletto MG, Gerlich WH, Heermann KH, Bardini R, Alberti A (1989). Identification of an attachment site for human liver plasma membranes on hepatitis B virus particles. Virology.

[32] Sobotta D, Sominskaya I, Jansons J, Meisel H, Schmitt S, Heermann KH (2000). Mapping of immunodominant B-cell epitopes and the human serum albumin-binding site in natural hepatitis B virus surface antigen of defined genosubtype. J Gen Virol..

[33] Wang HC, Huang W, Lai MD, Su IJ (2006). Hepatitis B virus pre-S mutants, endoplasmic reticulum stress and hepatocarcinogenesis. Cancer Sci.

[34] Huy TT, Ushijima H, Win KM, Luengrojanakul P, Shrestha PK, Zhong ZH (2003). High prevalence of hepatitis B virus pre-s mutant in countries where it is endemic and its relationship with genotype and chronicity. J Clin Microbiol.

[35] Chen CH, Changchien CS, Lee CM, Hung CH, Hu TH, Wang JH (2008). Combined mutations in pre-s/surface and core promoter/precore regions of hepatitis B virus increase the risk of hepatocellular carcinoma: a case-control study. J Infect Dis.

[36] Fang ZL, Sabin CA, Dong BQ, Wei SC, Chen QY, Fang KX (2008). Hepatitis B virus pre-S deletion mutations are a risk factor for hepatocellular carcinoma: a matched nested case-control study. J Gen Virol.

[37] Lin CL, Liu CH, Chen W, Huang WL, Chen PJ, Lai MY (2007). Association of pre-S deletion mutant of hepatitis B virus with risk of hepatocellular carcinoma. J Gastroenterol Hepatol.

[38] Chen BF, Liu CJ, Jow GM, Chen PJ, Kao JH, Chen DS (2006). High prevalence and mapping of pre-S deletion in hepatitis B virus carriers with progressive liver diseases. Gastroenterology.

[39] Chen CH, Hung CH, Lee CM, Hu TH, Wang JH, Wang JC (2007). Pre-S deletion and complex mutations of hepatitis B virus related to advanced liver disease in HBeAg-negative patients. Gastroenterology.

[40] Hsieh YH, Su IJ, Wang HC, Chang WW, Lei HY, Lai MD (2004). Pre-S mutant surface antigens in chronic hepatitis B virus infection induce oxidative stress and DNA damage. Carcinogenesis.

[41] Hsieh YH, Su IJ, Wang HC, Tsai JH, Huang YJ, Chang WW (2007). Hepatitis B virus pre-S2 mutant surface antigen induces degradation of cyclin-dependent kinase inhibitor p27Kip1 through c-Jun activation domain-binding protein 1. Mol Cancer Res.

[42] Su IJ, Wang HC, Wu HC, Huang WY (2008). Ground glass hepatocytes contain pre-S mutants and represent preneoplastic lesions in chronic hepatitis B virus infection. J Gastroenterol Hepatol.

[43] Wang HC, Wu HC, Chen CF, Fausto N, Lei HY, Su IJ (2003). Different types of ground glass hepatocytes in chronic hepatitis B virus infection contain specific pre-S mutants that may induce endoplasmic reticulum stress. Am J Pathol.

[44] Wang HC, Chang WT, Chang WW, Wu HC, Huang W, Lei HY (2005). Hepatitis B virus pre-S2 mutant upregulates cyclin a expression and induces nodular proliferation of hepatocytes. Hepatology.

[45] Belloni L, Pollicino T, De Nicola F, Guerrieri F, Raffa G, Fanciulli M (2009). Nuclear HBx binds the HBV minichromosome and modifies the epigenetic regulation of cccDNA function. Proc Natl Acad Sci U S A.

[46] Lucifora J, Arzberger S, Durantel D, Belloni L, Strubin M, Levrero M (2011). Hepatitis B virus X protein is essential to initiate and maintain virus replication after infection. J Hepatol.

[47] Levrero M, Zucman-Rossi J (2016). Mechanisms of HBV-induced hepatocellular carcinoma. J Hepatol.

[48] Gottlob K, Fulco M, Levrero M, Graessmann A (1998). The hepatitis B virus HBx protein inhibits caspase 3 activity. J Biol Chem.

[49] Han J, Yoo HY, Choi BH, Rho HM (2000). Selective transcriptional regulations in the human liver cell by hepatitis B viral X protein. Biochem Biophys Res Commun.

[50] Lucito R, Schneider RJ (1992). Hepatitis B virus X protein activates transcription factor NF-kappa B without a requirement for protein kinase C. J Virol.

[51] Iavarone M, Trabut JB, Delpuech O, Carnot F, Colombo M, Kremsdorf D (2003). Characterisation of hepatitis B virus X protein mutants in tumour and non-tumour liver cells using laser capture microdissection. J Hepatol.

[52] Ma NF, Lau SH, Hu L, Xie D, Wu J, Yang J (2008). COOH-terminal truncated HBV X protein plays key role in hepatocarcinogenesis. Clin Cancer Res.

[53] Astrovskaya I, Tork B, Mangul S, Westbrooks K, Mandoiu I, Balfe P (2011). Inferring viral quasispecies spectra from 454 pyrosequencing reads. BMC Bioinformatics.

[54] Ley TJ, Mardis ER, Ding L, Fulton B, McLellan MD, Chen K (2008). DNA sequencing of a cytogenetically normal acute myeloid leukaemia genome. Nature.

[55] Solmone M, Vincenti D, Prosperi MC, Bruselles A, Ippolito G, Capobianchi MR (2009). Use of massively parallel ultradeep pyrosequencing to characterize the genetic diversity of hepatitis B virus in drug-resistant and drug-naive patients and to detect minor variants in reverse transcriptase and hepatitis B S antigen. J Virol.

[56] Margeridon-Thermet S, Shulman NS, Ahmed A, Shahriar R, Liu T, Wang C (2009). Ultra-deep pyrosequencing of hepatitis B virus quasispecies from nucleoside and nucleotide reverse-transcriptase inhibitor (NRTI)-treated patients and NRTI-naive patients. J Infect Dis.

[57] Nishijima N, Marusawa H, Ueda Y, Takahashi K, Nasu A, Osaki Y (2012). Dynamics of hepatitis B virus quasispecies in association with nucleos(t)ide analogue treatment determined by ultra-deep sequencing. PLoS One.

[58] Scholz MB, Lo CC, Chain PS (2012). Next generation sequencing and bioinformatic bottlenecks: the current state of metagenomic data analysis. Curr Opin Biotechnol.

[59] Lin YY, Hsieh CH, Chen JH, Lu X, Kao JH, Chen PJ (2017). De novo assembly of highly polymorphic metagenomic data using in situ generated reference sequences and a novel BLAST-based assembly pipeline. BMC Bioinformatics.

[60] Guo Y, Ye F, Sheng Q, Clark T, Samuels DC (2014). Three-stage quality control strategies for DNA re-sequencing data. Brief Bioinform.

[61] Lin HJ, Lai CL, Lauder IJ, Wu PC, Lau TK, Fong MW (1991). Application of hepatitis B virus (HBV) DNA sequence polymorphisms to the study of HBV transmission. J Infect Dis.

[62] Osiowy C, Giles E, Tanaka Y, Mizokami M, Minuk GY (2006). Molecular evolution of hepatitis B virus over 25 years. J Virol.

[63] Sterneck M, Gunther S, Gerlach J, Naoumov NV, Santantonio T, Fischer L (1997). Hepatitis B virus sequence changes evolving in liver transplant recipients with fulminant hepatitis. J Hepatol.

[64] Conrad DF, Keebler JE, DePristo MA, Lindsay SJ, Zhang Y, Casals F (2011). Variation in genome-wide mutation rates within and between human families. Nat Genet.

[65] Bell TG, Kramvis A, Abdurakhmonov IY (2016). The study of hepatitis B virus using bioinformatics. Bioinformatics - updated features and applications: InTec.

[66] Liu WC, Lin CP, Cheng CP, Ho CH, Lan KL, Cheng JH (2016). Aligning to the sample-specific reference sequence to optimize the accuracy of next-generation sequencing analysis for hepatitis B virus. Hepatol Int.

[67] Bell TG, Yousif M, Kramvis A (2016). Bioinformatic curation and alignment of genotyped hepatitis B virus (HBV) sequence data from the GenBank public database. Spring.

[68] Liu WC, Wu IC, Lee YC, Lin CP, Cheng JH, Lin YJ (2017). Hepatocellular carcinoma-associated single-nucleotide variants and deletions identified by the use of genome-wide high-throughput analysis of hepatitis B virus. J Pathol.

[69] Fuentes Fajardo KV, Adams D, Program NCS, Mason CE, Sincan M, Tifft C (2012). Detecting false-positive signals in exome sequencing. Hum Mutat.

[70] Chen K, Wallis JW, McLellan MD, Larson DE, Kalicki JM, Pohl CS (2009). BreakDancer: an algorithm for high-resolution mapping of genomic structural variation. Nat Methods.

[71] Ye K, Schulz MH, Long Q, Apweiler R, Ning Z (2009). Pindel: a pattern growth approach to detect break points of large deletions and medium sized insertions from paired-end short reads. Bioinformatics.

[72] Sun R, Love MI, Zemojtel T, Emde AK, Chung HR, Vingron M (2012). Breakpointer: using local mapping artifacts to support sequence breakpoint discovery from single-end reads. Bioinformatics.

[73] Cheng CP, Lan KL, Liu WC, Chang TT, Tseng VS (2016). DeF-GPU: efficient and effective deletions finding in hepatitis B viral genomic DNA using a GPU architecture. Methods.

[74] Cheng JH, Liu WC, Chang TT, Hsieh SY, Tseng VS (2017). Detecting exact breakpoints of deletions with diversity in hepatitis B viral genomic DNA from next-generation sequencing data. Methods.

[75] Thoreson GR, Gayed BA, Chung PH, Raj GV (2014). Emerging therapies in castration resistant prostate cancer. Can J Urol.

[76] Sidhu K, Kapoor NR, Pandey V, Kumar V (2015). The “macro” world of microRNAs in hepatocellular carcinoma. Front Oncol.

[77] Oehler N, Volz T, Bhadra OD, Kah J, Allweiss L, Giersch K (2014). Binding of hepatitis B virus to its cellular receptor alters the expression profile of genes of bile acid metabolism. Hepatology.

[78] Watanabe Y, Yamamoto H, Oikawa R, Toyota M, Yamamoto M, Kokudo N (2015). DNA methylation at hepatitis B viral integrants is associated with methylation at flanking human genomic sequences. Genome Res.

[79] Su IJ, Wang LH, Hsieh WC, Wu HC, Teng CF, Tsai HW (2014). The emerging role of hepatitis B virus pre-S2 deletion mutant proteins in HBV tumorigenesis. J Biomed Sci.

[80] Wang Q, Xu Y, Zhou W, Zhong L, Wen Z, Yu H (2014). The viral oncoprotein HBx of hepatitis B virus promotes the growth of hepatocellular carcinoma through cooperating with the cellular oncoprotein RMP. Int J Biol Sci.

[81] Sung WK, Zheng H, Li S, Chen R, Liu X, Li Y (2012). Genome-wide survey of recurrent HBV integration in hepatocellular carcinoma. Nat Genet.

[82] Jain S, Chang TT, Chen S, Boldbaatar B, Clemens A, Lin SY (2015). Comprehensive DNA methylation analysis of hepatitis B virus genome in infected liver tissues. Sci Rep.

[83] Lauder IJ, Lin HJ, Lau JY, Siu TS, Lai CL (1993). The variability of the hepatitis B virus genome: statistical analysis and biological implications. Mol Biol Evol.

[84] Liang T, Chen EQ, Tang H (2013). Hepatitis B virus gene mutations and hepatocarcinogenesis. Asian Pac J Cancer Prev.

[85] Xu C, Zhou W, Wang Y, Qiao L (2014). Hepatitis B virus-induced hepatocellular carcinoma. Cancer Lett.

[86] Takahashi K, Akahane Y, Hino K, Ohta Y, Mishiro S (1998). Hepatitis B virus genomic sequence in the circulation of hepatocellular carcinoma patients: comparative analysis of 40 full-length isolates. Arch Virol.

[87] Miyake Y, Oda T, Li R, Sugiyama K (1996). A comparison of amino acid sequences of hepatitis B virus S gene in 46 children presenting various clinical features for immunoprophylaxis. Tohoku J Exp Med.

[88] Oon CJ, Chen WN (1998). Current aspects of hepatitis B surface antigen mutants in Singapore. J Viral Hepat.

[89] Oon CJ, Chen WN, Zhao Y, Teng SW, Leong AL (1999). Detection of hepatitis B surface antigen mutants and their integration in human hepatocellular carcinoma. Cancer Lett.

[90] Lee WP, Lan KH, Li CP, Chao Y, Lin HC, Lee SD (2012). Pro-apoptotic or anti-apoptotic property of X protein of hepatitis B virus is determined by phosphorylation at Ser31 by Akt. Arch Biochem Biophys.

[91] Huang Y, Tai AW, Tong S, Lok AS (2013). HBV core promoter mutations promote cellular proliferation through E2F1-mediated upregulation of S-phase kinase-associated protein 2 transcription. J Hepatol.

[92] Li H, Wu K, Tao K, Chen L, Zheng Q, Lu X (2012). Tim-3/galectin-9 signaling pathway mediates T-cell dysfunction and predicts poor prognosis in patients with hepatitis B virus-associated hepatocellular carcinoma. Hepatology.

[93] Wang F, Zhou H, Yang Y, Xia X, Sun Q, Luo J (2012). Hepatitis B virus X protein promotes the growth of hepatocellular carcinoma by modulation of the notch signaling pathway. Oncol Rep.

[94] Xu J, Liu H, Chen L, Wang S, Zhou L, Yun X (2012). Hepatitis B virus X protein confers resistance of hepatoma cells to anoikis by up-regulating and activating p21-activated kinase 1. Gastroenterology.

[95] Liu L, Li Y, Zhang S, Yu D, Zhu M (2012). Hepatitis B virus X protein mutant upregulates CENP-A expression in hepatoma cells. Oncol Rep.

[96] Sze KM, Chu GK, Lee JM, Ng IO (2013). C-terminal truncated hepatitis B virus x protein is associated with metastasis and enhances invasiveness by C-Jun/matrix metalloproteinase protein 10 activation in hepatocellular carcinoma. Hepatology.

[97] Yip WK, Cheng AS, Zhu R, Lung RW, Tsang DP, Lau SS (2011). Carboxyl-terminal truncated HBx regulates a distinct microRNA transcription program in hepatocellular carcinoma development. PLoS One.

[98] Chen Z, Tang J, Cai X, Huang Y, Gao Q, Liang L (2016). HBx mutations promote hepatoma cell migration through the Wnt/beta-catenin signaling pathway. Cancer Sci.

[99] Diao J, Khine AA, Sarangi F, Hsu E, Iorio C, Tibbles LA (2001). X protein of hepatitis B virus inhibits Fas-mediated apoptosis and is associated with up-regulation of the SAPK/JNK pathway. J Biol Chem.

[100] Elmore LW, Hancock AR, Chang SF, Wang XW, Chang S, Callahan CP (1997). Hepatitis B virus X protein and p53 tumor suppressor interactions in the modulation of apoptosis. Proc Natl Acad Sci U S A.

[101] Minor MM, Slagle BL (2014). Hepatitis B virus HBx protein interactions with the ubiquitin proteasome system. Viruses.

[102] Tang H, Delgermaa L, Huang F, Oishi N, Liu L, He F (2005). The transcriptional transactivation function of HBx protein is important for its augmentation role in hepatitis B virus replication. J Virol.

[103] Ito K, Tanaka Y, Orito E, Sugiyama M, Fujiwara K, Sugauchi F (2006). T1653 mutation in the box alpha increases the risk of hepatocellular carcinoma in patients with chronic hepatitis B virus genotype C infection. Clin Infect Dis.

[104] Lyu H, Lee D, Chung YH, Kim JA, Lee JH, Jin YJ (2013). Synergistic effects of A1896, T1653 and T1762/A1764 mutations in genotype c2 hepatitis B virus on development of hepatocellular carcinoma. J Viral Hepat.

[105] Tatsukawa M, Takaki A, Shiraha H, Koike K, Iwasaki Y, Kobashi H (2011). Hepatitis B virus core promoter mutations G1613A and C1653T are significantly associated with hepatocellular carcinoma in genotype C HBV-infected patients. BMC Cancer.

[106] Tong MJ, Blatt LM, Kao JH, Cheng JT, Corey WG (2007). Basal core promoter T1762/A1764 and precore A1896 gene mutations in hepatitis B surface antigen-positive hepatocellular carcinoma: a comparison with chronic carriers. Liver Int.

[107] Li MS, Lau TC, Chan SK, Wong CH, Ng PK, Sung JJ (2011). The G1613A mutation in the HBV genome affects HBeAg expression and viral replication through altered core promoter activity. PLoS One.

[108] Park YM, Jang JW, Yoo SH, Kim SH, Oh IM, Park SJ (2014). Combinations of eight key mutations in the X/preC region and genomic activity of hepatitis B virus are associated with hepatocellular carcinoma. J Viral Hepat.

[109] Cullen JM, Marion PL, Newbold JE (1989). A sequential histologic and immunohistochemical study of duck hepatitis B virus infection in Pekin ducks. Vet Pathol.

[110] Wu Y, Gan Y, Gao F, Zhao Z, Jin Y, Zhu Y (2014). Novel natural mutations in the hepatitis B virus reverse transcriptase domain associated with hepatocellular carcinoma. PLoS One.

[111] Li H, Jia J, Wang M, Wang H, Gu X, Fang M (2017). F221Y mutation in hepatitis B virus reverse transcriptase is associated with hepatocellular carcinoma prognosis following liver resection. Mol Med Rep.

